# Role of Gamma-Aminobutyric Acid in Plant Defense Response

**DOI:** 10.3390/metabo13060741

**Published:** 2023-06-10

**Authors:** Zhujuan Guo, Junqing Gong, Shuitian Luo, Yixin Zuo, Yingbai Shen

**Affiliations:** National Engineering Research Center of Tree Breeding and Ecological Restoration, College of Biological Sciences and Technology, Beijing Forestry University, No. 35, Qinghua East Road, Beijing 100083, China; guozhujuan@bjfu.edu.cn (Z.G.); gongjunqing2018@bjfu.edu.cn (J.G.); alice0521@bjfu.edu.cn (S.L.); zuoyx2020@bjfu.edu.cn (Y.Z.)

**Keywords:** gamma-aminobutyric acid, synthesis and metabolism, carbon and nitrogen metabolism, pH balance, antioxidant system, calcium signal

## Abstract

Gamma-aminobutyric acid (GABA) is a four-carbon non-protein amino acid that acts as a defense substance and a signaling molecule in various physiological processes, and which helps plants respond to biotic and abiotic stresses. This review focuses on the role of GABA’s synthetic and metabolic pathways in regulating primary plant metabolism, redistributing carbon and nitrogen resources, reducing the accumulation of reactive oxygen species, and improving plants’ tolerance of oxidative stress. This review also highlights the way in which GABA maintains intracellular pH homeostasis by acting as a buffer and activating H^+^-ATPase. In addition, calcium signals participate in the accumulation process of GABA under stress. Moreover, GABA also transmits calcium signals through receptors to trigger downstream signaling cascades. In conclusion, understanding the role of GABA in this defense response provides a theoretical basis for applying GABA in agriculture and forestry and feasible coping strategies for plants in complex and changeable environments.

## 1. Introduction

As the global climate changes, the environment in which plants live becomes increasingly complex. In the face of various biotic and abiotic stresses (drought, salt, high temperature, cold, etc.), plants develop different defense mechanisms [1,2,3]. Primary metabolism plays a role in plant defense mechanisms. Under stress, plants can reduce photosynthesis and enhance respiration to remobilize carbon and nitrogen resources [4]. In addition, pH homeostasis, reactive oxygen species (ROS), and Ca^2+^ are essential signaling molecules involved in plants’ responses to stress. Intracellular pH homeostasis is the basic cell activity required to maintain normal plant growth, regulate plant growth and development, and respond to environmental stress. ROS enhances resistance by inducing cell damage through oxidative properties to promote plant morphology and structural change. It can also interact with transcription factors to regulate the expression of defense-related genes [5]. Changes in the intracellular calcium concentration are the first step in the plant’s defense response. Calcium signals activate calmodulin and other calmodulin receptors, triggering protein phosphorylation and changes in gene expression patterns [6,7].

GABA is a class of four-carbon non-protein amino acids that exists widely in animals, plants, and microorganisms. It is used as an important inhibitory neurotransmitter in animals and is mainly concentrated in the central nervous systems of mammals [8]. GABA accumulates rapidly in plants under various stresses [9,10] and can directly participate in the plant defense response as a defensive substance and in the response to stress as a signaling molecule [11,12]. In this paper, we address the synthesis and metabolism of GABA in plants, focusing on GABA’s response to stress by participating in the carbon and nitrogen metabolisms and in the regulation of pH, the antioxidant system, and Ca^2+^ signaling.

## 2. Synthesis and Metabolism of GABA

The synthesis and metabolism of GABA in plants are mainly realized through a branch of the tricarboxylic acid (TCA) cycle, also known as the GABA shunt, which includes three key enzymes: glutamic acid decarboxylase (GAD), gamma-aminobutyric acid aminotransferase (GABA-T), and succinate semialdehyde dehydrogenase (SSADH) [13].

**Figure 1:**
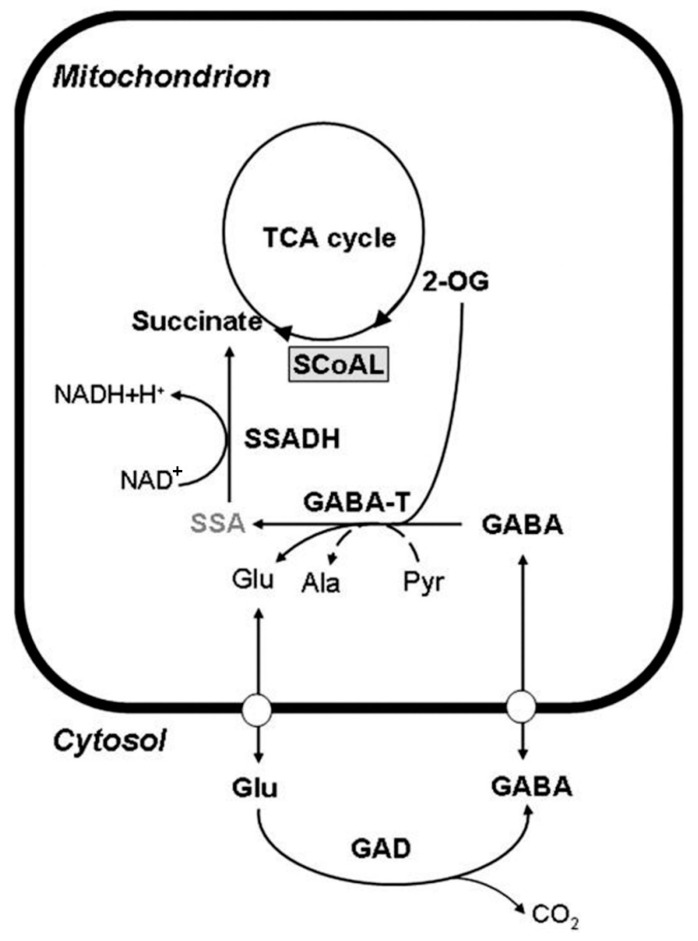
GABA metabolic pathways in higher plants [13]. Adapted from the publication, with permission.

Glutamic acid (Glu) in plants is decarboxylated to produce GABA under the catalysis of GAD in the cytoplasm. Subsequently, GABA in the cytoplasm can enter the mitochondrial matrix through GABA transporters on the mitochondrial membrane and is reversibly decomposed into succinic semialdehyde by GABA-T catalysis. On the one hand, the toxic intermediate SSA is transported out of the mitochondria to form in the cytosol GHB via the enzyme SSR. On the other hand, they can be reversibly catalyzed to form succinic acid by SSADH to participate in the tricarboxylic acid cycle (Figure 1). Studies have found that GABA in plants can also be transformed and formed through the degradation of polyamines. Putrescine, spermidine, and spermine are catalyzed by polyamine oxidase to form H_2_O_2_ and pyrroline, and the latter is catalyzed by pyrroline dehydrogenase to form GABA. However, the specific mechanism of this pathway remains unclear [14,15,16].

**Figure 2:**
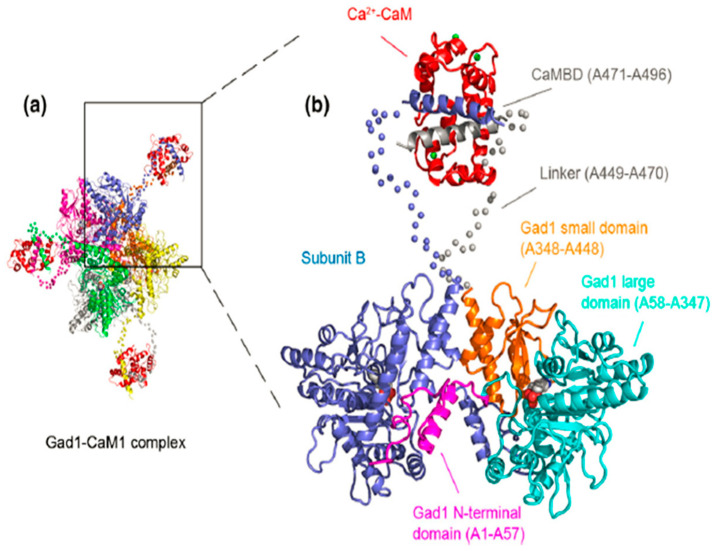
Domain organization of Gad1 and detailed view of the CaM-binding mode. Reprinted with permission from [18]. Copyright © Oxford University Press. (**a**) The diagram shows the SAXS model of Gad1-CaM1 complex. Each Gad1 subunit is a different color, and the CaM molecule is red. (**b**) Subunit B (blue) is depicted as a cartoon with the linker atoms as blue spheres. Subunit A is colour-coded by domain: N-terminal domain, magenta; large domain, cyan; small domain, orange; C-terminal linker, grey spheres; CaMBD, grey. CaM (red) is depicted as a cartoon with bound Ca^2+^ as green spheres. The cofactors of two adjacent active sites appear in space-fill mode (atom colours).

GAD is widely found in eukaryotes and prokaryotes belonging to pyridoxal 5′-phosphate compounds (PLP). Under the action of PLP and other cofactors, GAD can specifically catalyze the removal of carboxyl from intracellular glutamic acid to form GABA [17]. Typical GAD in plants usually exists in the form of dimers or hexamers; each monomer is composed of four typical domains, in which amino acid residues 1–57 constitute the N-terminal domain, which is mainly related to the formation and stability of GAD polymers. The large domain, composed of residues 58–347, contains a pyridoxal phospho-specific α/β folding structure and a cofactor binding site. The third small domain is composed of 348–448 amino acid residues, including three α helical structures and four β lamellar structures. The C-terminal consists of a pH-sensitive CaM-binding domain (CaMBD), which can sense changes in intracellular pH and regulate enzyme activity by binding with Ca^2+^/CaM in response to intracellular Ca^2+^ signals (Figure 2). The C-terminal domain of GAD has a self-inhibition ability, so the activity of GAD in plant cells remains low under normal physiological conditions. Studies have shown that the activity of GAD in plants is highest in the acidic pH range, and the optimal pH value is 5.8. GAD regulates enzyme activity by binding to intracellular Ca^2+^/CaM when the cell pH exceeds the normal physiological pH value.

**Figure 3:**
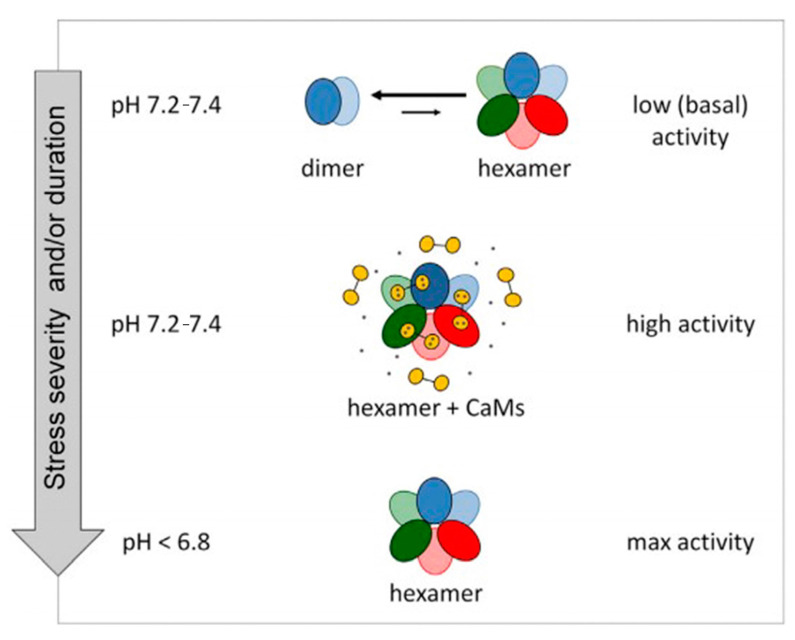
The effect of cytosolic pH on GAD multimerization. Reprinted with permission from [19], Copyright © Elsevier.

However, when the cell environment is lower than the physiological pH, GAD loses the ability to bind Ca^2+^/CaM, and pH mainly affects its activity [18]. GAD in *Arabidopsis thaliana* was analyzed by size exclusion chromatography, and it was found that GAD existed in dimer form under normal pH conditions. However, when pH drops or when Ca^2+^/CaM binds to the C-terminal, GAD changes from dimer to hexamer. This conformational change and the formation of polymers may be closely related to the change in activity [19] (Figure 3). In summary, plant cells perceive external stimuli through changes in intracellular pH and Ca^2+^ concentration and thus regulate GAD enzyme activity and cause changes in GABA metabolism.

The expression level of GAD and the content of GAD protein in the leaves of plants cultured under different nutritional conditions are significantly different, indicating that the regulation of the transcription level of the GAD encoding gene also plays an essential role in the synthesis of GABA [20,21]. The regulation of *GAD* expression in different tissues and organs of *Pinus pinaster* Ait. is related to the synthesis of GABA. It involves many physiological processes, such as hypocotyl and stem development [22]. In addition, GAD is a sensitive gene in the plant GABA pathway that is responsive to stress. GAD can respond specifically to different stresses (freezing, high temperature, water stress, salt, UV-B radiation, and mechanical stimulation). The expression levels of *GAD1* and *GAD5* in the above-ground part were slightly upregulated under all stress treatments, while *GAD3* and *GAD4* were significantly upregulated under salt, UV-B radiation, and water stress conditions [23]. After the expression of *GAD1* was inhibited, the accumulation of GABA was significantly reduced under high-temperature treatment [24]. Similarly, studies in rice have shown that the expressions of two GAD members in germinated seeds under hypoxia treatment were significantly upregulated 12 h later. The expression of GAD in barley seedlings was significantly upregulated after a long low-temperature treatment, resulting in a large accumulation of GABA [25]. In tomatoes, the upregulated expression of *GAD1* can increase the contents of GABA and glutamate to alleviate salt stress damage [20]. In cotton, *GAD6* plays an important role in responding to Cd^2+^ stress [26].

GABA-T is a critical enzyme involved in the GABA metabolic pathway, mainly located in the cytoplasm, plastid, and mitochondrial matrix. It can catalyze the reversible decomposition of GABA transported from the cytoplasm to mitochondria or plastid to form succinic semialdehyde [27]. At present, many kinds of GABA-T have been identified in plants. GABA-TP in *Arabidopsis thaliana* can use pyruvate as an amino receptor to catalyze the transamidation of GABA [28], while GABA-TK, another aminotransferase identified in other plants, uses α-ketoglutaric acid as an amino receptor, but the activity of this enzyme is low. GABA-TK found in tomatoes has specific activity and can use glyoxalic acid as an amino receptor [29]. These results indicate that the GABA metabolic pathway forms a complex network with other metabolic pathways involved in regulating various physiological processes, and the GABA-T function is specified in different plants. In *Arabidopsis thaliana*, *GABA-T* is encoded by a single copy gene and is widely expressed in a variety of tissues and organs [27]. The accumulation of GABA in *Arabidopsis thaliana* plants after *GABA-T* knockout resulted in sterility caused by pollen tube development obstruction, while other phenotypes were not abnormal [30]. Exogenous GABA treatment to *gaba-t* mutant resulted in decreased chlorophyll content, inhibited primary root growth, and decreased expression of cell-wall-related proteins. These results suggest that the *GABA-T* mutation may prevent GABA degradation, which would affect plants’ ability to grow normally [31]. In addition, similar to *GAD*, *GABA-T* expression is also induced by a variety of stress conditions [23,25,32], *GABA-T* overexpressed plants have been seen to be more sensitive to salt stress in mulberry [33]. The increase in GABA in the root and above-ground parts of *gaba-t* seedlings has been seen to be significantly higher than that of the wild type, indicating that the seedling is more sensitive to salt stress than the wild type. In this process, the up-regulation of the expression of multiple members of *GAD* caused by salt treatment may be the main reason for the massive synthesis of GABA, and the mutation of *GABA-T* leads to the inability to degrade excessive GABA in plants, resulting in excessive accumulation in various tissues. These results indicate that GABA-T and GAD are involved in the synthesis and metabolism of GABA in plants in response to stress [34,35].

SSADH is not only a key metabolic enzyme in the GABA branch but also an important enzyme connecting the GABA branch and the TCA cycle. The activity of SSADH in plants is affected by a variety of factors, among which NAD^+^, the key cofactor, can activate SSADH, while NADH can inhibit SSADH activity. Studies have shown that the lower the ratio of NAD^+^/NADH, the faster the decline of SSADH activity. When the ratio reaches 0.5, SSADH activity is almost completely lost. Furthermore, studies have shown that the activity of SSADH is regulated by ATP feedback. The mutation of the SSADH in *Arabidopsis thaliana* affects electron transport in mitochondria, suggesting that SSADH mainly functions in mitochondria [36]. Therefore, under various stress conditions, the normal NAD^+^/NADH level of cells may be changed by affecting the energy state of cells, resulting in the restriction or competitive inhibition of SSADH activity and ultimately indirectly affecting the metabolism of GABA [23,25,37]. Under salt stress, the expression and activity of SSADH increases, and the metabolism of succinate is affected by the GABA pathway to adapt to environmental changes [38].

## 3. GABA Is Involved in Plant Metabolism

The metabolic environment in the cell changes with changes in the external environment, resulting in the response and adaptation of metabolic pathways. Organelles receive information about changes in the extracellular environment and transmit information in the form of intracellular metabolites and energy levels [39]. Carbon and nitrogen metabolism are two major metabolic processes in plant cells. Carbon metabolism provides carbon sources and energy for nitrogen metabolism, while nitrogen metabolism provides enzymes and photosynthetic pigments for carbon metabolism. Both carbon and nitrogen metabolism require common reducing power, ATP, and a carbon skeleton. The balance and regulation of carbon and nitrogen metabolism are particularly important in plant growth, development and response to stress. Under stress, Glu is catalyzed by GAD to rapidly synthesize GABA, which serves as an intermediary of plant nitrogen metabolism, allowing it to temporarily store nitrogen. Meanwhile, GABA is able to re-participate in the TCA cycle through the formation of succinic semialdehyde and succinic acid. GABA can serve as the equilibrium point of carbon and nitrogen metabolism, linking energy metabolism with carbon (C) and nitrogen (N) metabolism [28,40,41].

Through the use of key enzyme coding gene mutants in the TCA cycle, it has been found that the upstream metabolic enzyme mutation related to the formation of succinic acid can significantly affect the activity of the GABA branch, while the downstream metabolic enzyme coding gene mutation of succinic acid has no effect on GABA metabolism [42,43,44]. In transgenic tomatoes, GABA branches can partially compensate for the normal function of the TCA cycle after the deletion and mutation of the succinyl-CoA ligase coding gene, and isotope technology assessment has indicated that GABA branches can provide a succinic acid source for the TCA cycle to maintain the normal operation of TCA in this process [45]. In citrus, endogenous levels of GABA and succinic acid rapidly increased and entered the TCA cycle after the application of exogenous GABA, indicating that exogenous GABA can enhance plant resistance to biological and abiotic stresses by activating the GABA branch and the TCA cycle to generate more energy [46]. These results confirm the mutual regulatory relationship between the TCA cycle and the GABA branch. In the cellular environment, the expression patterns of key genes in the TCA cycle are different from those in the GABA branch, but GAD and SSADH in the GABA branch are positively correlated with the expression of some genes in the TCA cycle. Therefore, the interaction between GABA branches and the TCA cycle may jointly affect the carbon metabolism process of plants, and GABA branches may play a more important role than the TCA cycle in maintaining the normal physiological state of plants under stress [47].

GABA may be involved in nitrogen metabolism by regulating Glu. Glu is considered one of the main components mediating the C:N ratio in plants [48]. It also plays an important role in amino acid metabolism. Most inorganic nitrogen is assimilated through the glutamine synthetase/glutamate synthetase (GS/GOGAT) pathway to produce glutamate, which is the starting point of most amino acid synthesis, including GABA. When the GAD function is changed, the level of Glu is greatly affected [28,49]. After four days of long-term nitrogen treatment of Arabidopsis leaves, the unreduced nitrogen source (KNO_3_) was transferred to reduced nitrogen (NH_4_Cl, NH_4_NO_3_, glutamate, or glutamine), and the gene expression of *GAD2* was upregulated, indicating the involvement of the GABA branch [50]. In addition, stress induces other Glu-related metabolic pathways. Under osmotic pressure, a large amount of Glu is converted to GABA and proline (Pro). It has been proven that Pro transporters, such as AtProT2 and LeProT1, can transport GABA and some other organic osmotic substances simultaneously. These results indicate that these substances might act together as osmotic regulators to maintain the cellular water potential balance [51,52]. Under salt stress, amino acids have been shown to accumulate in wheat, represented by proline. Pro is not only an osmotic protective agent but also plays important roles in removing free radicals externally and affecting ion homeostasis in cells. Besides proline, arginine and ornithine are also involved in wheat’s response to salt stress via Glu metabolism [53]. Under normal growth conditions, Glu is converted to methionine (Met), threonine (Thr), isoleucine (Ile), and lysine (Lys) through the aspartic acid family pathway (Asp-family pathway). However, under stress conditions, the expression of the bifunctional polypeptide LKR/SDH formed by lys-ketoglutarate reductase (LKR) and saccharine dehydrogenase (SDH), which are related to Lys metabolism, is upregulated. These enzymes can transform Lys into Glu and re-participate in stress-induced metabolic regulation [54]. Glu is also involved in a variety of plant-growth-related metabolic pathways [51]. Therefore, the regulation of the Glu metabolism via the GABA pathway may be an important mechanism for plants to adjust growth resources under adverse conditions.

**Figure 4:**
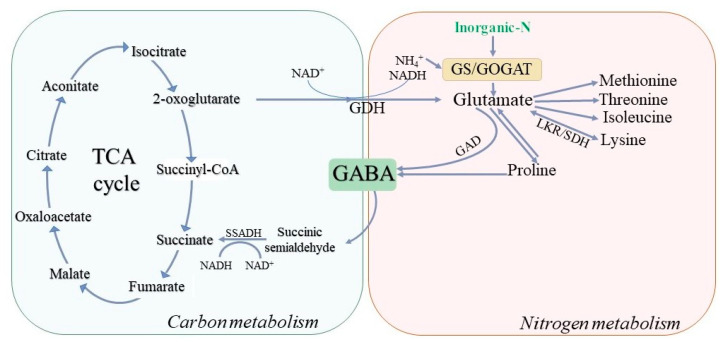
Diagrammatic representation of GABA regulating carbon and nitrogen metabolism. GABA plays an important role in the maintenance of C/N balance in plants. GABA is involved in carbon metabolism through TCA cycle and nitrogen metabolism by regulating glutamic acid. TCA cycle: tricarboxylic acid cycle; GABA: γ-aminobutyric acid; GAD: glutamate decarboxylase; SSADH: succinate semialdehyde dehydrogenase; GDH: glutamate dehydrogenase; GS/GOGAT: glutamine synthase/glutamate synthase; LKR/SDH: lys–ketoglutarate reductase/saccharopine dehydrogenase.

The GABA pathway is functionally irreplaceable in the cross-compartment regulation of primary metabolism, mitochondrial REDOX state and energy metabolism regulation [9,55]. Both protein folding and removal reactions in the endoplasmic reticulum (ER) depend on ATP, mitochondria are the main source of ATP in the cell, and the maintenance of mitochondrial energy metabolism and REDOX state is essential for the maintenance of efficient protein folding. The accumulation of unfolded proteins in the ER is called ER stress. Under stress conditions, an increase in calcium is activated, which leads to the accumulation of GABA through the activation of CaM domains of *GAD1, 2, 4*. ATP produced by the GABA branch in the electron transfer chain in mitochondria provides energy for the ER to relieve stress [56]. SSADH is responsible for supplying succinic acid to the TCA cycle through the oxidation of succinic semialdehyde (SSA). SSADH specifically oxidizes SSA and produces NADH via nicotinamide adenine dinucleotide (NAD^+^) for use in mitochondrial electron transport chains under salt stress [28]. SSADH is negatively regulated by NADH and ATP so that GABA branches can be regulated according to the REDOX and energy state of the cell [28,57]. GABA branches can provide succinic acid for the TCA cycle and compensate for the respiration process in mitochondria. When GAD or GABA-T is inhibited, the respiration rate of wheat leaves is significantly reduced, indicating that wheat respiration is more dependent on GABA branches [53]. These findings suggest that GABA may be involved in carbon and nitrogen metabolism through various pathways under stress, from the transcriptional level to enzyme activity regulation (Figure 4). However, the mechanism of GABA’s involvement in nitrogen absorption and assimilation remains unclear.

The GABA branch is crucial for reducing stress, controlling GABA metabolism in stressful situations, achieving a balance in the metabolism of carbon and nitrogen, and influencing other physiological processes such as plant growth and development [58,59,60]. By regulating the activity of GABA branches through GAD, GABA-T, and SSADH, plants improve their ability to endure stress. A significant contributing component to the buildup of GABA is GAD activation. Glutamate is transformed into GABA under the influence of GAD and takes part in plant defense processes as a substrate for its synthesis. Under cold stress, glutamate concentration also rises [61]. Seeds exposed to salt stress showed increased *GAD* expression, demonstrating the critical function GABA plays in carbon and nitrogen metabolism during seed germination [62]. Notably, GAD activity is similarly influenced by Ca^2+^ levels. Cytoplasmic Ca^2+^ levels can enhance plant tolerance to various stress situations by controlling GABA production, suggesting that GABA shunt is implicated in stress signal transduction in the plant system [63]. It has been observed that GABA-T activity is limited under stress conditions, determining that an accumulation of GABA is useful for the provision of anaplerotic succinate for the TCA cycle upon stress relief [64]. Alternatively, GABA-T can participate in the regulation of the occurrence of leaf senescence under stress conditions. Through characterization of Arabidopsis GABA transaminase knockout mutants *pop 2-1* and *pop 2-3*, it was found that on the one hand, *pop 2-1* and *pop 2-3* mutations rapidly reduced the stress-induced chlorophyll content and GABA content in leaves, as well as the activities of GABA-T and GAD, on the other hand, increased membrane ion leakage and MDA levels. This indicates that GABA-T can reduce the effectiveness of carbon dioxide in leaves by inhibiting carbon fixation, reducing electron transfer during photosynthesis and leading to ROS accumulation in chloroplasts, thereby triggering the process of leaf senescence [17]. The presence of SSADH can cause the GABA branch to produce NADH and provide an energy and carbon skeleton to the TCA cycle, regulating redox reactions under oxidative stress [65]. There is also increasing evidence showing that plants can improve their defense ability by regulating the TCA cycle through GABA branches. The enzymes which are diverted by the GABA shunt circumvent susceptible enzymes to oxidative stress. This susceptibility can decrease the efficiency of the TCA cycle; though the GABA shunt can reimburse this decrease in stressed plants. For instance, under low temperature, proteomics analysis of tea plants showed that exogenous GABA regulated carbon and nitrogen metabolism through the TCA cycle to enhance cold tolerance [66]. Under salt stress, the activity of key enzymes in the TCA cycle of wheat is inhibited, while the increase in GABA pathway activity provides mitochondria with alternative carbon sources to bypass salt-sensitive enzymes and enhance wheat respiration [53]. In addition, under salt treatment, GABA can also serve as a temporary nitrogen supply to reduce the excessive ammonium accumulated due to protein and amino acid catabolism and photorespiration [67]. Under low nitrogen conditions, the application of GABA in poplar significantly weakens the increase in leaf antioxidant activity. GABA may regulate the relative allocation of C and N in growth activities by reducing energy costs related to stress defense, indicating that GABA promotes poplar growth and adaptation by regulating C and N metabolic fluxes under nitrogen deficiency conditions [68]. Additionally, GABA can participate in osmotic regulation as an osmotic substance or promote the accumulation and regulation of osmotic products [69]. Under dehydration stress caused by high salt conditions, GABA plays a crucial role as an effective osmotic agent at the cellular level without harmful effects on plants [67]. Under oxidative and osmotic stress conditions, the large accumulation of GABA and Pro can serve as osmotic protectants, promoting cell synthesis and reducing cell degradation. Other studies have also reported on the defensive function of Pro in genetically modified crops, where its excessive production enhances tolerance to osmotic stress [58,59]. In addition to Pro, the response to osmotic stress also includes other amino acids; the overexpression of glutamine synthase, for example, enhances the tolerance of rice to osmotic stress. The change of osmotic pressure increases the content of amino acids due to the modification of gene expression encoding amino acid metabolic enzymes [70]. The metabolic pathway of the GABA pathway can be converted into these amino acids [12]. Exogenous application of GABA can maintain osmotic regulation by increasing soluble sugar and proline content, thereby improving the growth and productivity of black cumin under water deficit stress conditions [71]. Therefore, GABA maintains the normal physiological processes of plants in different ways under stress conditions.

## 4. GABA Regulates Intracellular pH

Dynamic changes in pH are induced by various stresses such as salinity, drought, hypoxia, flooding, fungal infection, ectomycorrhizal symbiosis, herbivore attack, or developmental processes such as growth, gravity responses, and processes associated with light sensing or stomatal opening. ABA can regulate stomatal movement through the alkalization of cytoplasm under salt and drought stress [72]. When a plant is attacked by a pathogen, a decrease in pH activates cell-wall-loosening enzymes that lead to cell wall loosening and degradation. Soon after, chitin mediates extracellular alkalinization to counteract the pH change caused by the pathogen, which is also a process of plant evolution [73]. Cytoplasmic pH balance is a prerequisite for normal plant growth and stress response. Basic cytoplasmic processes, such as biochemical reactions, protein stability, ion channel/transporter activity, and membrane transport have strict pH requirements [74,75]. The cytoplasmic pH of living plant cells needs to be maintained in the 7.0–7.5 range [76,77,78], and small changes in this range can alter metabolism or even cause cell death. The regulation mechanism of pH in plant cytoplasm can be basically divided into two kinds. One is a regulation mechanism based on metabolism, namely the biochemical pH-stat. This uses metabolites as pH buffers, such as carbonate and phosphate. There are also metabolic processes of proton production and consumption, such as carboxylation and decarboxylation of organic acids such as malic acid. The second regulatory mechanism is the biophysical pH-stat, the transmembrane transport of H^+^ between cytoplasm and exocytosis and vacuoles. This process is mainly carried out by various H^+^ pumps located on the membrane and also involves H^+^-coupled ion transporters and other ions to maintain the balance of electrochemical potential [79,80].

GABA can act as an effective pH buffer at high and low pH values. For example, GABA released from the root cytoplasm (assumed to be close to neutral) at high pH values will have a significant H^+^ efflux because GABA is an amphoteric molecule, with an amino group (pKa = 10.43) and a carboxyl group (pKa = 4.23). When pH is neutral, the amino group is protonated, and the carboxyl group is deprotonated, resulting in a neutral charge; at high pH, the amino group loses protons, so GABA has a negative charge. When a concentration of nutrients with buffer capacity (such as NH_4_^+^, H_2_PO_4_^−^, H_3_BO_3_, HCO_3_^−^, and CO_3_^2−^) is added to observe the change in pH value, it is found that a small amount, but a high concentration, of GABA will reduce the pH value to around 7.0. The experiment of Thwaites, D.T., and Kamran, M. also showed this result [81,82]. In addition, H^+^ is consumed when GAD catalyzes Glu to form GABA; it is thus speculated that GABA synthesis may be involved in regulating intracellular pH in plant cells. Real-time monitoring of GABA content and pH in asparagus cells has shown that the decrease in intracellular pH was accompanied by increased GABA content under the treatment of weak acid with cell membrane permeability. Moreover, after 45 s of treatment, the consumption of H^+^ in the GABA synthesis process accounted for 50% of the H^+^ contained in the weak acid. This indicates that the decrease in intracellular pH activates GAD and leads to an increase in GABA synthesis. The synthesis of GABA consumes part of the H^+^ [83]. Similarly, the change in intracellular pH in carrot cells and the change in GABA content in the process of ammonium assimilation are mutually regulated, and the synthesis of GABA can inhibit the intracellular acidification process in plant cells [84]. Under hypoxia stress, plant rhizosphere cells lack the final receptor molecule O_2_ of the respiratory electron transport chain, which leads to the suppression of aerobic respiration, while the activation of anaerobic respiration-related metabolism leads to cytoplasmic acidification. In this process, plant GAD is activated, resulting in a large amount of GABA synthesis and excessive H^+^ consumption, thus reducing the degree of damage to plant cells under hypoxia stress [85,86].

GABA can also influence intracellular pH by regulating the transmembrane transport of H^+^ through the activity of H^+^-ATPase. Under drought stress, GABA acts as a signaling molecule by binding to the GABA receptor (ALMTs) and regulates the activity of H^+^-ATPase through the 14-3-3 protein, thus regulating stomatal movement [87]. The H^+^ efflux of the mutant pop-5 with higher GABA content has been shown to be more obvious than that of *gad1* and *gad2*, with H^+^-ATPase involved in this process to improve the plant salt tolerance [34].

In conclusion GABA synthesis and metabolism can participate in the maintenance of the pH balance of plant cells under stress in a biochemical manner, while GABA accumulation under stress can activate H^+^-ATPase to maintain basic pH balance physically.

## 5. GABA Is Involved in the Antioxidant System

Reactive oxygen species (ROS) include free radicals (superoxide anion, O_2_^●−^; hydroperoxyl radical, HO_2_^●^; alkoxy radical, RO^●^; and hydroxyl radical, ^●^OH) and non-radical molecules (hydrogen peroxide, H_2_O_2_; and singlet oxygen, ^1^O_2_), and are the product of cell metabolism under environmental stress [88,89]. At low concentrations, they function as signaling molecules, which can activate defense response under stress. High concentrations of ROS can destroy proteins, nucleic acids, and lipids in cells as well as cell membranes, leading to metabolic disorders. When plants are subjected to abiotic stress, the balance between ROS production and the antioxidant defense system is disrupted, resulting in excessive accumulation of ROS, inducing oxidative stress in plants [90]. To this end, plants have evolved a set of antioxidant systems and defense mechanisms to reduce or even eliminate toxicity caused by ROS under stressful conditions, such as drought, salinity, heavy metals, and extreme temperatures [91]. The antioxidant system includes several antioxidant enzymes, such as superoxide dismutase (SOD), catalase (CAT), ascorbate peroxidase (APX), peroxidase (POD), dehydroascorbate reductase (DHAR) and mon-dehydroascorbate reductase (MDHAR), as well as some non-enzymatic molecules, such as carotenoids, flavonoids, total phenols and ascorbic acid (AsA) [92].

GABA accumulation is thought to reduce oxidative damage caused by ROS and thus increase tolerance to oxidative stress [93]. The plant cells decompose the accumulated GABA in the cell through the GABA branch, and the end product formed also acts as the substrate of the TCA cycle. When the GABA branch is blocked, the TCA cycle is inhibited, thus affecting the respiration in mitochondria and ultimately resulting in the accumulation of ROS [94]. Studies have shown that GABA can activate the antioxidant defense system under salt stress to reduce oxidative damage caused by ROS production [94]. The formation of GABA is promoted by activating the Ca^2+^/CaM pathway under UV-B radiation. However, the GABA pathway is blocked in *Arabidopsis thaliana* after *CaM*-related mutates, resulting in decreased ROS scavenging ability and resistance to UV-B radiation in seedlings and suggesting that the GABA pathway is involved in the regulation of the plant’s antioxidant pathway [93]. Many studies have shown that exogenous GABA treatment can maintain the REDOX state balance at the cell level by affecting the activity of antioxidant-related enzymes in plants to improve their resistance. Under various stress environments (chilling injury, hypoxia, salt, and high temperatures), plants pretreated with exogenous GABA reduced ROS and malondialdehyde contents by regulating the photosynthetic system of leaves and activating the antioxidant system to maintain the normal photosynthesis level and assimilation ability; thus, GABA has been shown to improve the adaptability of plants to adversity [95]. GABA also enhances plant growth by regulating membrane lipid peroxidation and ion homeostasis in muskmelon by stimulating the antioxidant system [96]. After exogenous GABA treatment, the activity of antioxidant enzymes in leaves was significantly enhanced, the accumulation of ROS was reduced, and the ability of seedlings to resist freezing, acid, and metal ions was significantly enhanced [97,98,99]. Tobacco accumulates a large amount of GABA and Pro under water stress. In vitro experiments have shown that GABA and Pro can remove ROS directly, and that GABA has a greater ability to remove O_2_^-^ and H_2_O_2_ than Pro. These results indicate that the accumulation of endogenous GABA in plants under stress is related to the antioxidant mechanism of plants [100].

**Figure 5:**
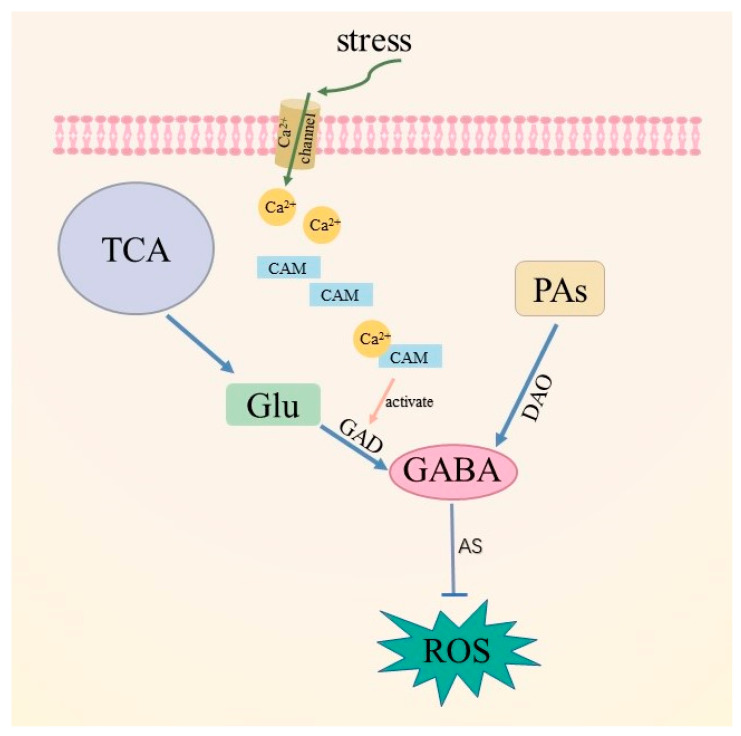
Schematic representation of a plant’s scavenger ROS by GABA. Under various stimuli, plants complete the accumulation of GABA through GABA branches and polyamine pathways, while GABA removes excess ROS through the antioxidant system. TCA: tricarboxylic acid cycle; PAs: polyamines; Glu: glutamic acid; GAD: glutamic acid decarboxylase; DAO: diamine oxidase; AS: antioxidant system; ROS: reactive oxygen species.

In addition to the GABA pathway, the metabolic pathways of polyamines (PAs) also participate in the accumulation of GABA. In *Vicia faba* L., the activities of GAD and diamine oxidase (DAO) were seen to be significantly increased and the accumulation of GABA significantly reduced after DAO activity was inhibited, suggesting that DAO is also involved in GABA synthesis under hypoxic conditions [101]. A large amount of GABA has been seen to be accumulated in the root system of soybean under salt stress; however, after DAO activity was inhibited, GABA content in the root system decreased by 40%, and polyamines increased by 54%, indicating that polyamines were involved in this process as precursor substances of GABA [102]. In plants, the polyamine pathway regulates enzymatic antioxidant activity, and PAs promote the accumulation of phenolic compounds and free proline to prevent oxidative damage. The interactions between GABA and PAs can regulate the production and removal of ROS, with the degradation process of polyamines accompanied by the production of ROS. Forming a kind of signaling substance, ROS and polyamines jointly regulate the signaling pathways related to plant stress resistance and defense [103,104]. Therefore, after polyamines degrade GABA, excess ROS can be removed. This mechanism ensures the signaling function of ROS, reduces the damage caused by ROS to plant cells, and maintains the normal REDOX level in cells (Figure 5).

## 6. GABA and Ca^2+^

Under different environmental stimuli, intracellular Ca^2+^ concentrations can rise more than tenfold in a matter of seconds, and this process can continue for some time [105]. In normal plant cells, the concentration of Ca^2+^ in the cytoplasm is 10,000 times lower than in the extracellular fluid and 100,000 times lower than the concentration of calcium ions in the organelles, creating an incentive for calcium ions to enter the cell via channel proteins as a second messenger [106]; this provides a guarantee for the role of calcium when it plays a role in the plant’s defense response [107]. Calcium signal transduction is usually activated in the following modes. Calcium ion changes are triggered by the cell’s perception of environmental changes, and calcium ion channels located on the cell membrane are activated to transport Ca^2+^ to the cytoplasm. This increases the concentration of calcium ions in the cytoplasm. Then, increased intracellular calcium ions activate Ca^2+^ binding proteins (CaBPs), such as calmodulin (CaM), calcium-dependent protein kinase (CDPK), and calmodulin-like protein (CML), which further decode calcium signals. At the same time, excess [Ca^2+^]_cyt_ is pumped out of the cell by a “pump out” mechanism, and [Ca^2+^]_cyt_ returns to its resting state [108].

The accumulation of GABA under biotic and abiotic stress is related to the regulation of Ca^2+^/CaM. When plants are subjected to temperature stress, drought, salinity, or mechanical treatment, the intracellular Ca^2+^ concentration increases, promoting the stimulation of Ca^2+^/CaM to GAD and causing the accumulation of GABA. For example, the accumulation of GABA in apples stored under controlled atmosphere conditions is due to the highly conserved CaMBD of *MdGAD1* and *MdGAD2*, whose activity and spectral properties are regulated by Ca^2+^/CaM and acidic pH [109]. GAD is highly sensitive to the Ca^2+^/CaM complex in rice bran; when the concentrations of Ca^2+^ and CaM are 200 μmol L^−1^ and 150 nmol L^−1^, respectively, the GAD of rice bran is significantly increased, by three times [110]. In the presence of fructose, Ca^2+^ and *BoCaM2* levels in Broccoli sprouts have been raised to form Ca^2+^/CaM complexes that bind to GAD. Similarly, exogenous calcium-activated GAD promoted GABA accumulation in shredded carrots and fresh-cut pears [111,112,113].

GABA transmits calcium signals through receptors. The process of transporting GABA into the cell by sodium-dependent GABA receptors in horizontal cells from the skate retina causes depolarization of membrane potential and activates L-type voltage-gated calcium channels to transport extracellular calcium ions into the cell, thus triggering the release of calcium ions from the intracellular calcium pool [114]. Similarly, GABA depolarizes granule cell precursors via GABA_A_ receptors, which leads to calcium increases in these cells [115]. In plants, ALMTs are considered receptors for GABA and anion channels [11]. ALMTs are thought to be receptors for GABA as well as anion channels that cause changes in membrane potential and activate calcium channels to transmit calcium signals [116,117]. For instance, 5 mM of GABA can trigger an action potential, but this will not cause Arabidopsis mesophyll to depolarize. This suggests that GABA acts similarly to glutamic acid when triggering downstream action potential signaling cascades through receptors or through direct action on calcium ion channels [118]. In addition, the calcium signal is also involved in the GABA transport process. ATP inhibits the GABA transport process in rat cortical astrocytes because the GABA transporter GAT is Na^+^ dependent. ATP induces an increase in intracellular Ca^2+^ concentration which in turn increases the activity of the Na^+^/Ca^2+^ exchanger (NCX), and the accumulation of Na^+^ in astrocytes then leads to the decrease of GAT activity [119]. In plants, changes in the intracellular calcium ion concentration regulate the anion channel ALMT12, which acts as a receptor for GABA, which in turn regulates pollen tube growth through its interaction with ALMT12 [120].

## 7. Conclusions

In recent years, an increasing number of studies have been conducted on the role of GABA in plant defense response. The synthesis of GABA uses Glu as a precursor and participates in the TCA cycle. Therefore, GABA can be used as a bridge to regulate carbon and nitrogen balance and participate in plant carbon and nitrogen metabolism resistance to various stresses. The accumulation of GABA reduces excess ROS in cells and improves the plant’s tolerance to oxidative stress. GABA can also act as a signaling molecule in response to various stresses. For example, GABA can maintain pH balance in plants by activating H^+^-ATPase and can affect Ca^2+^ transport through ALMTs, thus triggering downstream signaling cascades. Although progress has been made in the study of GABA in plants, there are still many questions to be explored, and the polyamine pathway synthesis of GABA has not been thoroughly studied. There are many Ca^2+^ channels in the plasma membrane of cells, but which calcium channel is activated by GABA remains unclear. Few studies have examined GABA’s signaling function through ROS.

Overall, the positive role of GABA in plant resistance was analyzed in detail in this paper, providing a theoretical basis for the application of GABA in agriculture and forestry. Exogenous GABA can be applied as an effective and sustainable strategy to improve plant tolerance under field conditions to combat complex and changeable environmental problems.
